# Prevalence and correlates of domestic violence against ever married women of reproductive age in India: changes during 2005-2015

**DOI:** 10.5249/jivr.v14i3.1697

**Published:** 2022-07

**Authors:** Masuma Yasmin, Katja Gillander Gådin, Eija Viitasara, Koustuv Dalal

**Affiliations:** ^*a*^ Independent Researcher, Kolkata, India.; ^*b*^ Div. of Public Health Sciences, School of Health Sciences, Mid Sweden University, Sundsvall, Sweden.

**Keywords:** Domestic violence, Prevalence, Women, India

## Abstract

**Background:**

The present study was conducted to estimate the prevalence rate of domestic violence against women and examine the socio-demographic status of the victims of domestic violence in India. The study also examined the differences in the prevalence of emotional, physical, and sexual violence against ever-married women over ten years (2005 - 2015) in all member states in India.

**Methods:**

The study used secondary data from NFHS-3 (2005-06) and NFHS-4 (2015-16). Cross-tabulation and multivariate analyses were performed. IBM SPSS V25 was used for data analysis.

**Results:**

The prevalence of domestic violence against married women in India in 2015 was as follows: emotional violence, 13%; physical violence, 28%; and sexual violence, 7%. Rajasthan achieved the highest decline in the prevalence of domestic violence against women over the last ten years since 2005. In addition, younger age, urban residence, lower level of education and lower socioeconomic status were essential predictors of domestic violence.

**Conclusions:**

There should be an improvement in female education, awareness generation regarding their rights, and better social support to reduce the prevalence of violence against women. In addition, engaging men in the fight against domestic violence could bring positive results.

## Introduction

Domestic Violence (DV) against women (VAW) is a challenging global public health issue affecting women and communities in high, middle, well as low-income countries.^1-5^ It occurs in many forms throughout the lifespan of women, such as sex-selective abortion, female infanticide, child abuse, incest, sexual harassment, rape, domestic violence, intimate partner violence (IPV), overwork, denial of healthcare, and abuse and neglecting older women. Women living as immigrants or refugees in another country, those living in orphanages, prisons, mental hospitals, rehabilitation centers, or other institutions, are often victimized.^6^ There have been various instances worldwide where rape, sexual slavery, torture and violence against women have been used as weapons of war. This arises from cultural and political situations of the countries that tend to create a distinction between the genders and result in the commodification of women.^7-10^

The Multi-Country Study on Women’s Health and Domestic Violence conducted by the WHO stated the widespread prevalence of violence against married/ ever-partnered women varied from 15% in Ethiopia to 71% in Japan.^3,4,11,12^ Violence against women has been found to have various far-reaching consequences, both familial and societal. This is an issue of significant concern because the victims of violence tend to suffer from severe long term health consequences, including physical, mental, sexual and reproductive problems.^3,13^ Several studies also indicate that domestic violence in the family may ultimately result in an increased risk of child abuse. The men who perpetrate domestic violence or IPV on their wives or partners are more likely to be abusers of children.^14^

Moreover, in the long run, men exposed to parental violence in their childhood tend to develop an accepting attitude towards wife-beating when they grow up. A study conducted in India revealed that the prevalence of wife-beating justification was 67% among the men who had witnessed parental violence in their childhood.^15^ Some studies also suggest that women who have been victims of IPV are more likely to practice substance abuse than women who have not experienced IPV.^16^

In India, violence against women poses a grave problem, as crimes against women continue to rise at an alarming rate.^17^ In India, like other low and middle-income countries, women are often thought of as the physically, emotionally, and intellectually weaker sex due to a patriarchal society. As a result of this gender bias and male dominance, women are often mistreated and victimized.^18,19^ They are denied their right to live and their freedom and human rights, provided by the Constitution of India.^19^ Women are subjected to various heinous crimes, such as foeticide, infanticide, child abuse, child marriage, honor killing, female genital mutilation, forced marriage, bride burning for dowry, wife-beating, sexual harassment at homes and workplaces, rape, acid attack, forced prostitution and medical neglect.^13,18-20^ However, this critical issue has gained the limelight in India only after the legislation (2005) against domestic violence, known as the Protection of Women from Domestic Violence Act.^1,13,18^

India, being a land of diversity, has various communities that are distinct in geography, language, culture, religion, and economy. Crimes against women are grossly underreported in many areas,^1,17^ mainly due to the fear of the social stigma associated with the victims and their families.^21^ There also exist variations among the states due to differences in socioeconomic development and women’s status.^1,22^ The National Crimes Record Bureau (NCRB) reported a total of 378277 crimes against women in India in 2018, the highest in Uttar Pradesh (59445 crimes) and the lowest in Nagaland (75 crimes), among the Indian states.^23^ A previous national study indicated that DV was a severe problem in India based on 2005 statistics. There is a strong association between prevalence rates and socioeconomic background. In addition, the husband's controlling behavior is also one of the predictors of domestic violence against women.^12^ After a decade, it is interesting to know how women are being victimized by their husbands/ partners. The current study would greatly help understand the predictors of domestic violence against women in India. The state-wise representation of data could help policy-makers to adopt DV prevention strategies, which would improve the lives of women and children in the long run. Understanding the predictors of DV and how they have changed through times is crucial to formulate the preventive strategies against DV in India. However, it still lacks of assessment on these changes in India.

Using a national representative sample from all over India (2015-2016), the present study was conducted to estimate the prevalence rate of domestic violence against women, to examine the socio-demographic status of the victims of domestic violence in India and also to find out the association between prevalence rate and socio-demographic status. The study also examined the differences in the prevalence of emotional, physical, and sexual violence against ever-married women over ten years (2005 - 2015) in all member states in India.

## Methods 

The study used secondary data from National Family Health Survey (NFHS) round three and four. NFHS-3 was conducted in 2005-06 and NFHS-4 was conducted in 2015-16. 

NFHS uses the Indian population census as a sampling base and follows a uniform sample design procedure using probability proportional to population size (PPS). NFHS employs two stages sampling techniques for rural areas and three stages sampling techniques for urban areas. Rural sample selections were made in two stages: PPS villages were selected as primary sampling units (PSUs). Then from each PSU, NFHS randomly selected the households. Urban sample selections were performed in three stages: using PPS municipality wards were selected as PSUs. The second stage, census enumeration blocks (CEB) were randomly selected from each PSU. The third stage, from each CEB, NFHS randomly selected households. 

NFHS-3 included 109 041 residential households. In total, 124 385 individual women of reproductive age (15 -49 years) participated in the survey (response rate 95%). NFHS-4 included 628892 residential households, where a total of 351625 women of reproductive age (94.5% response rate) participated in the survey. A more detailed description of the sampling and data collection procedure is available elsewhere (http://rchiips.org/NFHS/sub_report.shtml). 

One woman of reproductive age was selected from each household as per ethical committee guidelines. The NFHS-3 and NFHS-4 were conducted using the same methodology. As the population numbers increased and PPS has changed along with different administrative areas, two surveys have no chance to include the same CEB after ten years. However, there is no information on whether the same individual from NFHS-3 was followed up after ten years in the NFHS-4, as the respondents could be different. 

The NFHS – 3 and -4 questionnaires conducted a detailed survey on women's background, reproductive history, family planning, fertility preferences, antenatal and delivery care, domestic violence, and several other demographic and health-related questions, including child health, husband health and family issues. The current study has focused on domestic violence and the socio-demographic variables. 

**Dependent Variables**

Domestic violence against women was defined as whether the respondent was ever exposed to emotional, physical, and sexual violence by their husbands/partners. 

Emotional violence: husband ever (1) humiliated her; (2) ever threatened her with harm; and (3) ever insulted her or made her feel bad.

Physical violence: husband ever (1) pushed her, shaken her, or thrown something at her; (2) ever slapped her; (3) ever punched her with his fist or something harmful; (4) ever kicked or dragged her; (5) spouse ever tried to strangle or burn her, and (6) ever threatened or attacked her with a knife, gun, or other weapons.

Sexual violence: husband ever (1) physically forced sex when not wanted and (2) ever forced other sexual acts when not wanted.

**Independent variables**

Age (15-19, 20-24, 25-29, 30-34, 35-39, 40-44, 45-49), residency (rural/urban), educational level (no education, primary, secondary and higher), religion, economic status, Current working status (Yes, No) and working pattern (employed all year round, employed seasonal, employed occasionally) were used as independent variables. Seasonal work means employed for a particular season, say during monsoon, for work in the paddy field. Occasional employment is not dependent on any season or factor. Occasional workers are rarely employed in any work field.

There was nine Religion in the questionnaire. However, after preliminary frequency estimation, the current study has used Hindu, Muslim, and Other religions. Other religions consist of Christian, Sikhs, Buddhists, Jain, Jews, Parsi, and others. Economic status was assessed by the wealth index, which was used as a proxy of income and considered all major household economic elements and assets.^12^

The wealth index measured the economic status, a validated and well-utilized measurement in various demographic and health-related surveys in different countries.^12^ The wealth index is widely used to measure participants' economic status and helps determine the equity of health programs. One of its main objectives is to measure the ability to pay for health services and distribute health services among the poor. The wealth index is a composite measure of the cumulative living standard of a household. It is calculated from the household's ownership of selected assets, such as radio, television and bicycle; materials used for building house; types of water supply and sanitation facilities. A generated statistical procedure known as the principal components analysis is used, which places individual households on a continuous scale of relative wealth. The scale of relative wealth is standardized in relation to a standard normal distribution with a mean of zero and a standard deviation of one. Finally, the standardized scores are used to create groups that define wealth quintiles as: poorest, poorer, middle, richer and richest. Rutstein and Johnson (2004) introduced the wealth index used in India, and it includes any item that may reflect socioeconomic status, including household assets, utility services, and country-specific items.^24^

The current study has also considered domestic violence-related injuries. The following four questions were included in the questionnaires: i) ever had bruises; ii) ever had injury, sprain, or dislocations; iii) ever had wounds, broken bones, broken teeth, or other serious injuries; and iv) ever had severe burns because of husband’s violent acts. A comparison between 2005 and 2015 was performed.

Each of the dependent variables was 0 if the respondent did not experience any of the quoted events and as 1 if the respondent experienced at least one of the quoted events. The coding method was the same for all three types of domestic violence. 

Telangana, a member state in India, was newly created mainly due to administrative reasons after the 2005 survey. Also, the union territories of India were not included in the previous survey (2005). Therefore, in the latest 2015 survey, the comparable data for Telangana and Union Territories on domestic violence are missing.

**Statistical Analysis**

The cross-tabulation, including χ2 test, was used to examine the proportional difference of domestic violence by socio-demographic characteristics. Multi-variate logistic regression was used to predict domestic violence exposure by respondent's socio-demographic characteristics. Odds ratios with 95% CI was used as a statistical measure to determine the association between dependent and independent variables. IBM SPSS V25 was used for data analysis. Statistical significance was considered at P <.05.

**Ethical permission**

The current study has used secondary data and does not need any ethical permission. However, as per the survey protocol, ethical approval was sought from the Institutional Ethical Review Board (Ref. no./IRB/NFHS-4/01_1/2015) of the IIPS, Mumbai, India. The NFHS-3 and NFHS-4 have strictly followed the WHO ethical and safety recommendations for DV research for data collection to ensure women respondents' safety and generate full disclosure of actual violence scenarios. More information is available elsewhere.^12,15^

## Results

***National level***

The prevalence of domestic violence against married women in India in 2015 was as follows: emotional violence, 13%; physical violence, 28%; and sexual violence, 7%. During 2005 the prevalence was 14%, 31% and 8% for emotional, physical and sexual violence, respectively.

**Figure 1:**
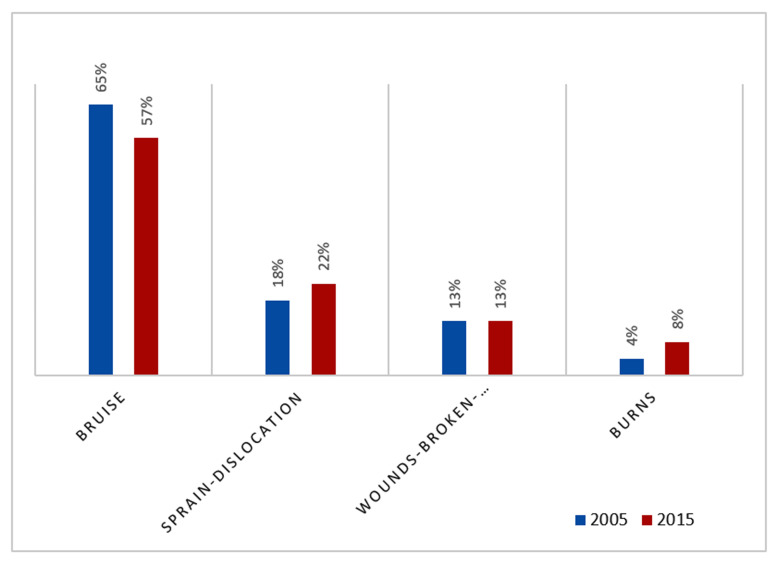
Domestic violence related injuries (2005 and 2015).

The severity of violence-related injuries is presented in Figure 1 for both 2005 and 2015. 

***State-wise comparison***

Table 1 indicates that in the year 2015, among the Indian states, Tamil Nadu (22%) had the highest prevalence of emotional violence against women, followed by Bihar (21%) and Andhra Pradesh (19%). On the other hand, Sikkim (3%) had the lowest prevalence of emotional violence, followed by Himachal Pradesh (4%), Goa (5%) and Uttarakhand (5%). 

**Table 1:** Number of Women in Each State (n) and Proportion within Each State Exposed to Domestic Violence (Percentage of n) in 2005 and 2015.

States	Emotional Violence (Percentage of n)			Physical Violence (Percentage of n)			Sexual Violence (Percentage of n)		
	2005	2015	Change	2005	2015	Change	2005	2015	Change
**Andaman & Nicobar Islands**		7			16			2	
**Andhra Pradesh**	12	19	7	32	43	11	10	6	-4
**Arunachal Pradesh**	17	16	-1	36	28	-8	4	7	3
**Assam**	14	11	-3	36	24	-12	14	5	-9
**Bihar**	23	21	-2	58	44	-14	21	14	-7
**Chandigarh**		7			20			5	
**Chhattisgarh**	13	16	3	31	36	5	7	7	0
**Dadra & Nagar Haveli**		15			30			4	
**Daman and Diu**		12			19			6	
**Delhi**	5	12	7	19	30	11	2	6	4
**Goa**	12	5	-7	17	12	-5	3	1	-2
**Gujarat**	19	12	-7	26	19	-7	8	5	-3
**Haryana**	10	13	3	28	32	4	8	9	1
**Himachal Pradesh**	3	4	1	6	5	-1	2	2	0
**Jammu and Kashmir**	9	10	1	12	10	-2	4	3	-1
**Jharkhand**	18	10	-8	35	32	-3	12	8	-4
**Karnataka**	8	12	4	20	20	0	4	6	2
**Kerala**	10	10	0	16	14	-2	5	4	-1
**Lakshadweep**		3			7			3	
**Madhya Pradesh**	23	12	-11	42	32	-10	11	8	-3
**Maharashtra**	15	10	-5	27	23	-4	2	3	1
**Manipur**	13	14	1	41	53	12	14	14	0
**Meghalaya**	7	12	5	12	24	12	2	4	2
**Mizoram**	11	10	-1	22	16	-6	2	3	1
**Nagaland**	12	10	-2	14	11	-3	3	6	3
**Odisha**	19	12	-7	33	34	1	14	9	-5
**Puducherry**		19			32			4	
**Punjab**	11	7	-4	25	20	-5	7	5	-2
**Rajasthan**	23	9	-14	41	25	-16	20	4	-16
**Sikkim**	10	3	-7	14	2	-12	5	1	-4
**Tamil Nadu**	18	22	4	44	41	-3	4	8	4
**Telangana**		22			43			7	
**Tripura**	22	13	-9	40	27	-13	19	9	-10
**Uttar Pradesh**	15	14	-1	43	36	-7	9	8	-1
**Uttarakhand**	10	5	-5	28	12	-16	6	3	-3
**West Bengal**	11	14	3	31	32	1	19	9	-10

Manipur (53%) had the highest prevalence of physical violence against women, followed by Bihar (44%), Andhra Pradesh (43%) and Tamil Nadu (41%). On the other hand, Sikkim (2%) had the lowest prevalence of physical violence, followed by Himachal Pradesh (5%) and Jammu and Kashmir (10%). 

Manipur and Bihar (both 14%) had the highest prevalence of sexual violence against women. On the other hand, Sikkim and Goa (both 1%) had the lowest prevalence of sexual violence, followed by Himachal Pradesh (2%).

Over the last ten years, since 2005, the Indian state of Rajasthan had achieved the highest decline in the prevalence of emotional violence (-14%), physical violence (-16%) and sexual violence (-16%) against women. Uttarakhand also reported a decline of 16% in the prevalence of physical violence. However, the North-Eastern states of Manipur and Meghalaya recorded an increase of 12% each in the prevalence of physical violence against women. 

***DV in the Union Territories***

Table 1 indicates that in the year 2015, among the union territories, Telangana (22%) had the highest prevalence of emotional violence against women, followed by Puducherry (19%), whereas Lakshadweep (3%) had the lowest prevalence. Similarly, Telangana (43%) also has the highest prevalence of physical violence against women, whereas Lakshadweep (7%) has the lowest prevalence. On the other hand, the highest prevalence of sexual violence against women is found in Telangana (7%) and the lowest in Andaman and Nicobar Islands (2%). 

***Demographic characteristics and Socioeconomic indicators (bi-variate analysis)***

Table 2 denotes that among the study participants, the proportions of women experiencing emotional and physical violence increase as the age of respondents increases. However, an inverse trend is observed for sexual violence; younger women are proportionally more sexually abused than older women. The prevalence of violence is found to be higher among the rural women (emotional violence, 13%; physical violence, 30%; and sexual violence, 7%), when compared to the urban women (emotional violence, 12%; physical violence, 24%; and sexual violence, 5%). Moreover, the prevalence of all three types of violence has decreased with the increase in educational status of the women. The proportions of Hindu women experiencing violence is higher (emotional violence, 13%; physical violence, 30%; and sexual violence, 7%), when compared to Muslim women (emotional violence, 13%; physical violence, 24%; and sexual violence, 6%). However, the prevalence of violence is found to be lowest in women belonging to other religions (emotional violence, 10%; physical violence, 23%; and sexual violence, 6%). 

**Table 2:** Number of Women in Each Category (n) and Proportion within Each Category Exposed to Domestic Violence (Percentage of n) in Terms of Demographic Characteristics in 2005 and 2015.

Variables	N		Emotional Violence (Percentage of n)			Physical Violence (Percentage of n)			Sexual Violence (Percentage of n)		
	2005	2015	2005	2015	Change	2005	2015	Change	2005	2015	Change
**Age (years)**			P<0.001	P<0.001		P<0.001	P<0.001		P<0.001	P=0.272	
**15-19**	3029	1642	12	12	0	25	20	-5	11	7	-4
**20-24**	10729	8847	13	11	-2	29	26	-3	9	7	-2
**25-29**	14974	13970	14	12	-2	31	27	-4	9	7	-2
**30-34**	14398	13598	14	13	-1	32	30	-2	8	7	-1
**35-39**	11827	11402	15	13	-2	32	29	-3	8	7	-1
**40-44**	8480	8677	15	13	-2	31	29	-2	7	7	0
**45-49**	5993	7877	15	14	-1	30	30	0	7	6	-1
**Residential area**			P<0.001	P<0.001		P<0.001	P<0.001		P<0.001	P<0.001	
**Urban**	30500	19469	13	12	-1	28	24	-4	7	5	-2
**Rural**	38928	46544	15	13	-2	34	30	-4	10	7	-3
**Education**			P<0.001	P<0.001		P<0.001	P<0.001		P<0.001	P<0.001	
**No education**	27529	22028	18	17	-1	42	37	-5	11	9	-2
**Primary**	10733	9669	17	14	-3	36	33	-3	10	8	-2
**Secondary**	25129	28187	11	11	0	23	23	0	6	5	-1
**Higher**	6030	6129	6	6	0	9	13	4	3	3	0
**Religion**			P<0.001	P<0.001		P<0.001	P<0.001		P<0.001	P<0.001	
**Hindu**	51619	49546	14	13	-1	32	30	-2	9	7	-2
**Muslim**	8594	8614	15	13	-2	35	24	-11	11	6	-5
**Others**	5709	7853	12	10	-2	21	23	2	4	6	2
**Economic status**			P<0.001	P<0.001		P<0.001	P<0.001		P<0.001	P<0.001	
**Poorest**	9728	12838	21	18	-3	46	41	-5	14	11	-3
**Poorer**	11111	13992	19	15	-4	42	33	-9	12	8	-4
**Middle**	13540	13790	16	13	-3	36	28	-8	10	7	-3
**Richer**	16039	13142	13	10	-3	30	23	-7	7	5	-2
**Richest**	19014	12251	8	7	-1	15	16	1	4	3	-1
**Respondent currently working**			P<0.001	P<0.001		P<0.001	P<0.001		P<0.001	P<0.001	
**No **	43736	49355	12	11	-1	28	26	-2	8	6	-2
**Yes**	25574	16658	18	17	-1	37	36	-1	10	9	-1
**Respondent employed all year/ Seasonal**			P=0.197	P<0.05		P<0.001	P<0.001		P<0.001	P<0.001	
**All year**	18915	12165	18	16	-2	36	33	-3	9	9	0
**Seasonal**	9345	8436	19	18	-1	42	41	-1	11	10	-1
**Occasional**	1254	1066	19	17	-2	40	36	-4	11	10	-1

X^2^ Significance Level

Table 2 also indicates that the prevalence of all three types of violence decreased with the increase in the economic status of the study participants. The women belonging to the higher socioeconomic status are less exposed to violence in India. However, the prevalence of violence is found to be higher among the women currently working (emotional violence, 17%; physical violence, 36%; and sexual violence, 9%), when compared to the women currently not working (emotional violence, 11%; physical violence, 26%; and sexual violence, 6%). In addition to this, the proportions of seasonally employed women experiencing violence is found to be higher (emotional violence, 18%; physical violence, 41%; and sexual violence, 10%), when compared to the women who are employed all year (emotional violence, 16%; physical violence, 33%; and sexual violence, 9%).

During the last 10 years (2005 -2015), the prevalence of all three types of violence against women has decreased or remained the same in all India's socio-demographic groups. However, an increase of 2% each has been observed in the prevalence of physical and emotional violence among women from other religions, including Christians, Sikhs, and Buddhists. Moreover, the prevalence of physical violence has increased by 4% among the women who have achieved higher education and by 1% among the women belonging to the richest economic quintile. 

***Demographic characteristics and Socioeconomic indicators (Multivariate Logistic Regression Analysis)***

In the recent survey (2015), younger married women are more likely to be victims of sexual violence in India than older married women. As indicated by the adjusted Odds Ratio (OR), urban women are more likely to be victims of all three types of violence in both surveys. The likelihood of experiencing all three types of violence decreases with the increase in the educational status of the women. Hindu women are more likely to experience physical violence than their peers from other religions. However, Hindu and Muslim women are more likely to experience emotional violence than their peers from other religions (Table 3).

**Table 3:** Number of Women in Each Category (n), Adjusted Odds Ratio (OR) for Exposure to Domestic Violence, and Confidence Intervals (CIs) of OR in Terms of Demographic Characteristics in 2005 and 2015.

Variables	N		Emotional Violence OR (CI)		Physical Violence OR (CI)		Sexual Violence OR (CI)	
	2005	2015	2005	2015	2005	2015	2005	2015
**Age (years)**								
**15-19**	3029	1642	0.72 (0.59-0.88) ***	1.01 (0.74-1.39)	0.61 (0.52-0.72) ***	0.65 (0.49-0.85)	1.40 (1.11-1.78) ***	1.32 (0.88-1.98)
**20-24**	10729	8847	0.90 (0.79-1.03)	0.98 (0.83-1.15)	0.92 (0.82-1.02)	1.003 (0.88-1.14)	1.25 (1.05-1.49)	1.55 (1.27-1.91)***
**25-29**	14974	13970	0.97 (0.86-1.09)	0.95 (0.84-1.09)	1.09 (0.98-1.19)	0.97 (0.87-1.07)	1.22 (1.04-1.44)	1.39 (1.17-1.65)***
**30-34**	14398	13598	0.95 (0.84-1.07)	0.99 (0.88-1.12)	1.08 (0.98-1.19)	1.08 (0.98-1.19)	1.18 (1.00-1.38)	1.20 (1.02-1.42)*
**35-39**	11827	11402	1.06 (0.94-1.19)	0.99 (0.87-1.12)	1.09 (0.99-1.21)	0.98 (0.88-1.08)	1.21 (1.03-1.43)	1.10 (0.93-1.30)
**40-44**	8480	8677	1.04 (0.92-1.18)	0.98 (0.86-1.11)	1.08 (0.97-1.19)	1.08 (0.98-1.19)	1.12 (0.94-1.33)	1.09 (0.91-1.30)
**45-49**	5993	7877	1	1	1	1	1	1
**Residential area **								
**Urban**	30500	19469	1.46 (1.35-1.58) ***	1.38 (1.25-1.52) ***	1.64 (1.53-1.74) ***	1.32 (1.22-1.43) ***	1.29 (1.17-1.43) ***	1.33 (1.17-1.52) ***
**Rural**	38928	46544	1	1	1	1	1	1
**Education**								
**No education**	27529	22028	2.62 (2.16-3.18) ***	1.99 (1.62-2.45) ***	3.74 (3.20-4.37) ***	2.47 (2.11-2.89) ***	2.32 (1.75-3.08) ***	2.52 (1.88-3.39) ***
**Primary**	10733	9669	2.95 (2.42-3.60) ***	1.89 (1.53-2.34) ***	3.67 (3.13-4.31) ***	2.25 (1.92-2.65) ***	2.49 (1.88-3.33) ***	2.30 (1.70-3.12) ***
**Secondary**	25129	28187	2.21 (1.84-2.67) ***	1.71 (1.41-2.09) ***	2.65 (2.28-3.08) ***	1.84 (1.58-2.13) ***	2.00 (1.52-2.63) ***	1.99 (1.49-2.64) ***
**Higher**	6030	6129	1	1	1	1	1	1
**Religion**								
**Hindu**	51619	49546	0.91 (0.79-1.04)	1.20 (1.08-1.34) ***	0.87 (0.77-0.97)	1.35 (1.24-1.47) ***	1.11 (0.92-1.35)	1.11 (0.97-1.28)
**Muslim**	8594	8614	0.94 (0.79-1.11)	1.34 (1.14-1.58) ***	0.84 (0.73-0.96)	0.99 (0.87-1.14)	1.41 (1.13-1.76) ***	1.02 (0.82-1.27)
**Others**	5709	7853	0.76 (0.64-0.90)	1	0.53 (0.46-0.61)	1	1	1
**Economic status **								
**Poorest**	9728	12838	2.19(1.89-2.53) ***	2.37 (1.97-2.85) ***	3.19 (2.83-3.59) ***	2.99 (2.60-3.46) ***	2.71 (2.22-3.31) ***	2.22 (1.73-2.84) ***
**Poorer**	11111	13992	1.97 (1.72-2.27) ***	2.08 (1.74-2.49) ***	2.83 (2.52-3.17) ***	2.42 (2.11-2.78) ***	2.17 (1.79-2.64) ***	1.72 (1.35-2.19) ***
**Middle**	13540	13790	1.59 (1.39-1.82) ***	1.92 (1.61-2.29) ***	2.28 (2.05-2.54) ***	2.03 (1.77-2.32) ***	1.93 (1.60-2.33) ***	1.65 (1.30-2.09) ***
**Richer**	16039	13142	1.31 (1.15-1.48) ***	1.48 (1.24-1.76) ***	1.91 (1.72-2.11) ***	1.75 (1.53-2.00) ***	1.61 (1.34-1.93) ***	1.37 (1.08-1.74) ***
**Richest**	19014	12251	1	1	1	1	1	1
**Respondent currently working **								
**No**	43736	49355	1.08 (0.99-1.18)	0.88 (0.80-0.96) ***	1.01 (0.94-1.09)	0.91 (0.85-0.97) ***	1.23 (1.09-1.37) ***	0.90 (0.80-1.01)
**Yes**	25574	16658	1	1	1	1	1	1
**Respondent employed all year/ Seasonal **								
**All year**	18915	12165	1.01 (0.87-1.18)	0.99 (0.83-1.17)	0.88 (0.78-0.99)	0.96 (0.84-1.09)	0.99 (0.82-1.20)	0.91 (0.73-1.12)
**Seasonal**	9345	8436	0.93 (0.79-1.09)	1.01 (0.85-1.19)	0.93 (0.82-1.06)	1.12 (0.98-1.28)	0.99 (0.82-1.21)	0.92 (0.74-1.14)
**Occasional**	1254	1066	1	1	1	1	1	1

***P<0.001, *P<0.05

The adjusted ORs indicate that the poorest women are 2 to 3 times more likely to experience all three types of violence than the richest women. It is also observed that the working respondents are less likely to be victims of physical violence compared to their non-working peers. 

## Discussion

The present study using national representative data (2015 study) from the 36 member states and union territories of India indicated the prevalence of emotional violence against women is 13%, physical violence is 28% and sexual violence is 7%. A previous study conducted in India, using the NFHS-3 data (2005), stated the prevalence of emotional violence against women was 14%, physical violence to be 31% and sexual violence to be 8%.^12^ As is evident from the statistics above, the prevalence of all three types of violence against women in India has decreased in the last 10 years. 

The government of India passed the Protection of Women from Domestic Violence Act 2005 for effective protection of women who are subjected to domestic violence and the act was brought into force in 2006. Protection officers, police officers and service providers were further trained to enforce the law effectively. The duties of shelter homes and medical facilities were laid down clearly, and preventive steps were taken. The prevalence of violence against women in India may have decreased in the last 10 years due to this act.^13,18^

The current study has highlighted important demographic characteristics and socioeconomic indicators that act as risk factors for domestic violence against women in India. Demographic characteristics such as age, residential area and educational status have influenced the occurrence of violence against women. The prevalence of emotional and physical violence increased with age, while sexual violence decreased with age. A similar trend has been observed in several other studies.^3,12,25,26^ However, a study conducted in China found that women’s age was not associated with intimate partner violence.^27^ Another study conducted in Ethiopia stated that women aged 25-29 years were more likely to experience physical violence while women aged 30-34 years were more likely to experience sexual violence.^11^

The present study indicated that the women living in the urban areas were more likely to be exposed to domestic violence than those living in the rural areas. This finding contradicts a study conducted in Pakistan or with the WHO multi-country study, which stated that rural women with high parity were more likely to be victims of violence.^3,4,11,12,28^ However, in a study conducted in Iran, it was observed that women living in urban areas had the highest prevalence of physical violence.^29^ In addition, a study in Eastern India revealed that living in urban areas was a risk factor for violence.^1^ It could be due to due to several factors such as hard and struggling living style in urban areas, urban stress, rural-urban migration related survival hardship. However, without specific explorative studies it’d be difficult to explain the actual reasons.

In a study conducted in Bangladesh, the qualitative findings stated that improving women's educational status was an important factor that reduced their exposure to domestic violence.^25^ The present study also revealed similar findings and the prevalence of violence was found to decrease with the increase in the educational status of the women. Similar phenomenon was indicated in several other studies.^1,3,11,12,26,28-33^ The reason could be that education improves women's status, gives them a good position in the family, increases their personal freedom and their will to defend themselves, gives them decision-making power, and reduces dependence. Moreover, education empowers women by indirectly increasing their earning potential. However, our study findings contradict a study conducted among South-Asian women residing in the US, which found that the occurrence of violence was higher among the highly educated South-Asian women than among the US population.^34^ Another study conducted in China stated that higher educational status was strongly associated with an increased risk of emotional violence against women.^27^

In another study conducted in Bangladesh, religion was found to be a significant predictor of domestic violence against women, with Muslim women experiencing a higher risk of violence than their Hindu counterparts.^30^ One more study in Bangladesh stated that Muslim women were at higher risk of being victimized during pregnancy.^35^ This contradicts our present study, which revealed that Hindu women were more likely to face physical and sexual violence, whereas Muslim women were more likely to face emotional violence. It could be due to majority of the Hindu religion in India and so the respondents. However, without specific explorative studies it’d be difficult to explain the actual reasons.

The prevalence of violence was found to decrease with the increase in the economic status of the respondents. Higher socioeconomic status was associated with a lower risk of violence against women. Similar findings were revealed in several other studies.^1,3,12,25,28-31,35^ However, the study conducted in China observed that economic inequality between husband and wife was a more important determinant of domestic violence against women than the overall familial, economic status.^27^ Therefore, further study may be warranted in the Indian context exploring socioeconomic inequality among the spouses and domestic violence against women.

A study conducted in Bangladesh stated that the women who contributed their earnings to cover household expenses were significantly more likely to report violence than those who contributed very little or none of their earnings.^25^ Similarly, in the present study, it was found that currently, working women were at higher risk of being abused than non-working women. A similar trend was observed in several other studies.^3,11,12,26,30,32^ The probable reason could be the male patriarchy in Indian society, which entitles men to be the sole breadwinners of the family and gives them superior rights, privileges, and authority over women.^1,12^ Due to these entrenched gender inequalities in India, women are often victimized by men when they step out of the home to work and seek independence. 

The study has unique advantages. It has used nationally representative samples, which offers a generalized result for India, including the prevalence of DV. Both the surveys have used the same sampling techniques and questionnaires. Therefore, the comparison of findings during the last ten years are most likely to be the actual scenario of DV in Indian member states. This could encourage the policymakers for state-wise DV prevention strategies. To the best of the authors' knowledge, this is the first study that compared the three types of DV in all the member states and union territories. 

Previous studies have argued that NFHS underestimate the extent of domestic violence against women compared with other surveys such as the WHO’s multi-country survey on violence against women and other specialized surveys on violence against women.^1,12,13,18,33^ Therefore, the prevalence presented in the current study based on NFHS could underestimate DV in India. However, as the NFHS had used the same data collection mechanism and questionnaires, the comparison between NFHS-3 (2005) and NFHS-4 (2015) is highly authentic. Finally, the cross-sectional study design does not draw the causal inference. NFHS-3 and NFHS -4 did not question the same respondents. Therefore, a longitudinal study design is highly warranted to establish causal links. 

## Conclusion

Violence against women is strongly related to the status struggles women tend to experience in Indian society. Male dominance and patriarchy lead to marital conflicts and power imbalance, resulting in the normalization of violence against women. There should be an improvement in female education, awareness generation regarding their rights, and better social support to reduce the prevalence of violence against women. In addition, engaging men in the fight against domestic violence could bring positive results. From a young age, men should be taught to respect women and treat them as equals. The current study first compared DV prevalence over ten years and showed a decline in DV prevalence in India. However, the rate is significantly higher compared to OECD countries. Indian health ministry, along with education and social ministry, needs to set up new and/or revised strategies using a 'bottom up' approach involving all relevant stakeholders to control DV significantly. 
